# Contributions of Auditory Nerve Density and Synchrony to Speech Understanding in Older Cochlear Implant Users

**DOI:** 10.1007/s10162-025-00984-3

**Published:** 2025-04-04

**Authors:** Kara C. Schvartz-Leyzac, Carolyn M. McClaskey, James W. Dias, Bryan E. Pfingst, Kelly C. Harris

**Affiliations:** 1 Department of Otolaryngology-Head & Neck Surgery, Medical University of South Carolina, Charleston, SC 29425 USA; 2 Department of Otolaryngology-Head & Neck Surgery, Medical Science II, Kresge Hearing Research Institute, University of Michigan, Ann Arbor, MI 48109 USA

**Keywords:** Cochlear implant, Neural health, Aging, Electrophysiology

## Abstract

**Purpose:**

The majority of adult cochlear implant (CI) recipients are over the age of 65, and previous research in non-implanted older adults shows that auditory nerve (AN) pathophysiology contributes to senescent declines in speech understanding. However, age-related changes to AN structure and function have not yet been explored as a contributory factor to poorer speech understanding outcomes in older CI users. Here, we explore how estimates of AN disengagement (i.e., AN density) and dyssynchrony in CI users contribute to poorer speech recognition performance observed in older CI users.

**Methods:**

We examined electrically evoked compound action potentials (ECAPs) in 47 adult (Male = 25) CI recipients. We measured the interphase gap (IPG) effect for the amplitude-growth function (AGF) slope and the N1-P2 interpeak latency as independent metrics of AN density and dyssynchrony, respectively.

**Results:**

Estimates of AN density and dyssynchrony worsen with increasing age in older CI listeners. These measures were not significantly correlated with one another, but were independently related to speech recognition in noise performance. Lower ECAP IPG effect values (lower density of AN fibers) are observed in older CI users. Longer N1-P2 interpeak latency values (poorer neural synchrony) are also observed in older CI users. When controlling for listener age, poorer AN dyssynchrony contributes to declines in speech-recognition-in-noise performance in CI users.

**Conclusion:**

These results suggest that AN dyssynchrony rather than density is the primary contributing factor to age-related declines in speech understanding in CI users. These results have important implications for better understanding neural contributions to speech understanding in adult CI users.

## Introduction

Cochlear implants (CIs) are widely considered to be the most successful neural prostheses. They have partially restored hearing to over one million recipients worldwide[3]. However, post-operative speech-recognition abilities vary widely across CI recipients[4–7] and several factors appear to contribute to this variability. Recent research has shown that one such factor is the condition of the auditory nerve (AN), which is expected given that the electrode array directly stimulates the AN. Animal models and human post-mortem temporal bone studies have shown that the density or total number of SGNs (the cell bodies of the auditory nerve) is lower in ears with hearing loss compared to normal-hearing controls, decreases with age, and can also vary within an ear from base to apex in the cochlea [8–12].

In animals, suprathreshold ECAP measures such as peak amplitude, slope of the amplitude growth function (AGF), and the recovery function, correlate with the total SGN density [11–15], thereby allowing for assessment of AN health in living human CI users. In humans, studies have shown that the total SGN count and/or density, as assessed using post-mortem temporal bone studies and by non-invasive measures such as the electrically evoked compound action potential (ECAP), positively correlates with post-operative speech recognition in adult CI recipients [16–19]. Importantly, ECAP measures such as the interphase gap (IPG) effect of the peak amplitude or slope also correlate with SGN density in animals and speech recognition in humans but are not influenced by electrode placement; ECAP measures using a fixed IPG duration are influenced by medial–lateral distance[1, 2]. Taken together, these results suggest that lower SGN counts are associated with poorer speech understanding in cochlear implanted adults, and specific ECAP measures that uniquely reflect SGN density can be used to help assess this relationship.

In non-implanted ears with normal hearing (NH) or hearing loss (HL), characteristics of AN function also correlate with speech recognition; more specifically, age-related changes to AN pathophysiology help to account for senescent declines in more complex tasks of speech understanding [20–22]. Likewise, simple measures of speech understanding (e.g., speech recognition in quiet) are not consistently associated with the age of the CI recipient, but aging more often negatively impacts complex measures of speech understanding in cochlear implanted adults (e.g., speech understanding in noise, and temporally compressed or rapid speech) [4, 7, 23, 24]. While several studies point to cognitive contributions of age-related declines in complex speech processing in CI recipients, the condition of the AN is also important to consider given the association between AN function and speech recognition in the aging non-implanted population[20–22]. Because the majority of adult recipients are over the age of 65 [25, 26], it is important to consider how age interacts with AN function in CI recipients to help further explain disparities in adult CI recipients’ outcomes.

**Fig. 1:**
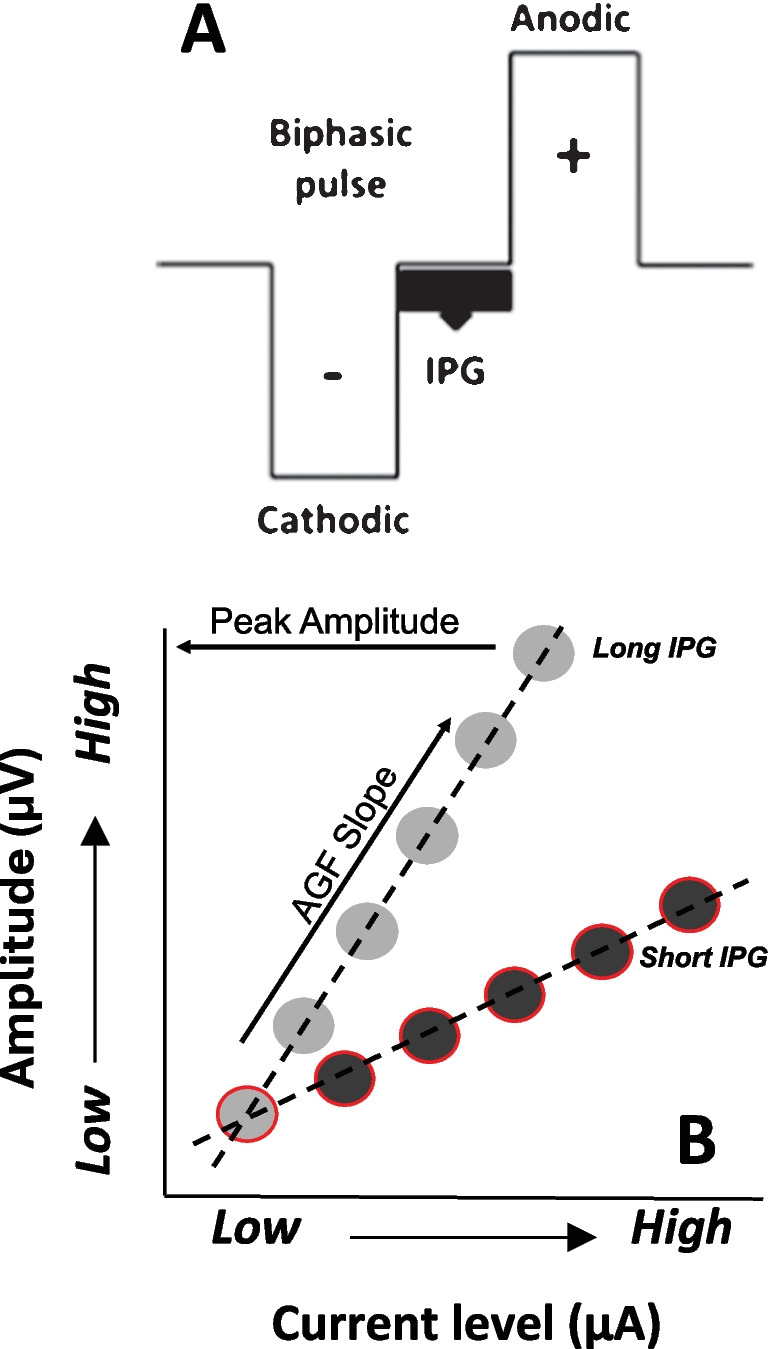
(**A**) and (**B**) show a schematic of the electrically evoked compound action potential (ECAP) interphase gap (IPG) effect for linear slope. (**A**) is a biphasic electrical pulse standardly used in cochlear implant (CI) stimulation methods. It consists of a leading cathodic (negative) phase followed by an anodic (positive phase); in between the cathodic and anodic phases is a specified duration of neutral charge, known as the IPG. The IPG can be manipulated when measuring ECAPs in CI stimulation. (**B**) shows how increasing the IPG duration changes ECAP characteristics known to correlate with spiral ganglion neural (SGN) density (amplitude and linear slope). As shown here, the amplitude of the N1-P2 ECAP response grows by increasing the current level used for stimulation. When increasing the IPG but holding all other factors constant, both the amplitude and linear slope of the ECAP amplitude growth function (AGF) increase. The increase in amplitude or linear slope as a result of increasing the IPG is associated with SGN survival

In our previous study, we showed that age-related declines in speech recognition can be partially explained by AN disengagement and dyssynchrony[20]. In non-implanted ears, AN disengagement could be due to reduced number of AN fibers, reduced endocochlear potential, and/or cochlear synaptopathy. In CI recipients, we hypothesize that AN disengagement is largely attributed to a reduced number of AN fibers, since a reduced endococochlear potential and/or cochlear synaptopathy should not affect AN disengagement in electrically stimulated ears. Disengagement in CI users can be estimated using ECAP measures. As noted above[12], large ECAP amplitudes suggest higher SGN density adjacent to the stimulated electrode. However medial–lateral distance also contributes to ECAP amplitude[1, 2]. Therefore, we propose that the IPG Effect for ECAP AGF slope (Fig. 1) represents AN disengagement in humans, as this metric has been shown to reflect the density of surviving SGN cells in cochlear implanted animals yet is not significantly influenced by electrode location in humans[1, 2, 12]. Given that the other causes of AN disengagement (related to hair-cell pathology) are largely irrelevant in the case of CI stimulation, we will henceforth use AN density to describe disengagement in CI users.

**Fig. 2:**
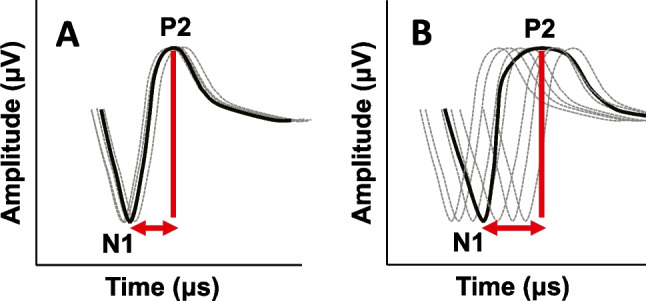
Schematic of the ECAP N1-P2 interpeak latency measure. Panel **A** shows a response with low trial-by-trial temporal jitter and a highly synchronous response where the individual trials are synchronous and do not differ significantly in latency or width. Panel **B** shows a response with greater dyssynchrony and high temporal jitter between individual trials, resulting in both a longer N1-P2 latency as well as a wider response overall. Thick black lines indicate the trial-averaged N1P2 response and grey dotted lines indicate individual trials. Interpeak latency is calculated as the duration between the N1 and P2 peaks and is marked with a red line and arrow

Similar to work in the non-implanted aging population[20], we define AN dyssynchrony as the reduced synchrony in the summated activity of AN fibers. This previous study by Harris et al.[20] used a phase locking value (PLV) measure (also known as ‘inter-trial coherence’) via the acoustically stimulated compound action potential (CAP). This measure is accomplished by examining individual (non-averaged) CAP tracings, and then calculating the intertrial phase coherence; better AN synchrony is equal to stronger intertrial phase coherence, while poorer AN synchrony is equal to weaker intertrial phase coherence. Recent studies have explored methods (including PLV) to capture AN dyssynchrony in CI users [27, 28], but these methods are complex and, in the case of PLV, potentially flawed due to limitations of ECAP recording parameters such as inadequate sampling rate and sampling window length which limit frequency resolution and difficulty in recording single-trial ECAPs; these limited recording parameters result in an imprecise PLV calculation in CI users, making it clinically difficult to assess. Alternatively, the current study explores a novel ECAP measure (N1-P2 interpeak latency — Fig. 2) to capture AN dyssynchrony in CI users. N1-P2 interpeak latency is analogous to half-width reported in non-implanted older adults [22]. If the individual peak latencies for the ECAP recordings vary significantly from trial to trial, then the averaged ECAP waveform would result in a wider N1-P2 interpeak latency which is analogous with a wider half-width, lower PLV, and greater dyssynchrony. Conversely, if individual peak latencies for the ECAP recordings are more similar from trial to trial, then the averaged ECAP waveform would result in a narrower N1-P2 interpeak latency, which is analogous with a narrower half-width, higher PLV, and better synchrony. In non-implanted listeners, age-related AN dyssynchrony is suggested to be a result of AN myelin degeneration[29] and therefore in electrically stimulated ears, we also hypothesize that AN dyssynchrony is also due to myelin degeneration.

The present study examined ECAP metrics in cochlear implanted adults to precisely describe how aging affects peripheral neural health function (AN density and synchrony), and how age-related changes to AN function affect speech understanding in background noise. We used a simple, multi-metric ECAP approach to estimate AN density and synchrony in a population of adult CI recipients; we predict that AN density and synchrony both worsen with increased age, as is seen in non-implanted listeners, and that both factors help to account for age-related declines in complex speech understanding in cochlear implanted adults.

## Materials and Methods

Data were collected across two different institutions (Medical University of South Carolina and the University of Michigan). This study was approved by the institutional review board (IRB) and both institutions where data were collected, and participants agreed through the informed consent process at each institution.

### Participants

Participants (N = 47) were adult cochlear implant (CI) recipients with adult-onset hearing loss (Ages 43–88; Male = 25). Twenty-five participants were recruited from one tertiary medical center and another 22 were recruited from a second tertiary medical center; data were collected at two different locations as a result of a change in workplace of the first author while the study was in progress. All participants were implanted with Cochlear™ implant systems (CI24RECA, CI422, CI512, CI612, CI622) and had at least 6 months’ experience with their CI. Twenty participants had been bilaterally implanted by the time of data collection. For the purpose of this study, only the first ear implanted was included in the analysis. As outlined below, not all of the 47 participants were included for all measures in the study.

### Electrically Evoked Compound Action Potentials

Electrically evoked compound action potentials (ECAPs) were measured in all participants (N = 47) and assessed using methods previously reported [16, 30]. Briefly, ECAPs were measured on each electrode, using forward-masking artifact reduction method [31, 32]. ECAPs were not measured on any electrode that was deactivated in a clinical map. ECAPs were collected using Cochlear Corporation CustomSound EP Version 6.0 or later. Default parameters were used for most participants (80 pps stimulation rate, 20 kHz sampling rate, 50–100 sweeps per average). The recording delay (default = 122 µs) and gain (50 dB) were sometimes adjusted in order to improve morphology and visualization of the N1 peak. Prior to ECAP measures, behavioral threshold and maximum comfort levels (MCLs) were assessed on each electrode in order to prevent patient discomfort when testing.

ECAPs were measured by stimulating from just below behavioral threshold to the patient’s MCL, for each electrode in an ascending order; for each electrode, the increase in current step was 5 clinical units (CUs). Clinical units were converted to µA as follows: 

1 $$\mu A=17.5\ast100(CU/255)$$

where *µA* represents current in units of microamps and “CU” represents arbitrary clinical units specified in the Cochlear software. ECAP responses represent the averaged waveform across all sweeps (50 individual recordings). All N1-P2 amplitude values below the noise floor of 5 µV were excluded.

We examined two ECAP metrics: the interphase gap (IPG) effect for the ECAP amplitude-growth function (AGF) slope and the N1-P2 interpeak latency at the peak-amplitude response. The IPG effect for the ECAP AGF slope was assessed using methods largely consistent with those reported previously[33]. Briefly, ECAP AGFs were recorded on each electrode using a 7 and 30 µs IPG. Prior to slope calculation, all µA values were converted to charge (nC = µA* phase duration in µs) depending on pulse width used for recording (25 or 37 µs, which was determined based on the participants clinical map). Different pulse width durations are required across CI recipients to stay within compliance limitations which are unique for each individual. For each electrode and IPG duration, the AGF was linearized by approximating the slope of the linear region using the gradient() function in MATLAB, and systematically removing the points that deviated by more than 20% of this slope[1, 2, 14, 33]. A linear model (*y* = *y0* + *ax*) was fit to the remaining points and the resulting slope was calculated. In all cases, the linear fit was statistically significant and produced an *R*^2^ of 0.90 or higher. The change in slope as a function of the IPG (the *IPG Effect*) was calculated for each electrode within each ear by subtracting the fitted AGF slope for the 7 µs IPG stimulus from that for the 30 µs IPG stimulus. The average IPG effect was calculated by taking the mean value across all electrodes within each ear. A schematic of the ECAP IPG Effect measures is shown in Fig. 1.

The N1-P2 interpeak latency value was calculated as follows. For each ECAP waveform on each electrode, a custom MATLAB program was used to measure N1 and P2 latencies at the peak-amplitude level, which is the ECAP recording measured at the patient’s MCL. N1-P2 interpeak latency was then calculated by subtracting N1 latency from P2 latency. The custom MATLAB program first identified the peaks within a given range specified by the examiner and fell within the expected latency range for the leading negative peak (N1) (150 to 300 µs) to the following positive peak (P2) (400–600 µs). The examiner was blinded to the demographics (i.e., Age and speech recognition score) for each participant when analyzing ECAP responses. The average N1-P2 interpeak latency for each participant was then obtained by calculating the mean value across all electrodes within an ear at the highest current level (nC). A schematic for the N1-P2 interpeak latency measure is shown in Fig. 2. We expect to observe longer (higher) N1-P2 interpeak latency for an averaged response when each individual recording was temporally less consistent (i.e., reflecting greater AN dyssynchrony); conversely, a shorter (lower) N1-P2 interpeak latency for an averaged response would be observed when each individual recording was temporally more consistent (greater AN synchrony).

### Computerized Tomography

Post-operative imaging was completed using advanced CT methods to better assess the position of the electrode array within the cochlea and to determine if ECAP measures representing AN health were influenced by electrode position. These data were obtained for only 17 of the 47 participants in this study. Due to data collection and processing performed over several years at different institutions, the current study uses two different methods of CT processing, necessitated by different standard clinical care and/or research practices between the University of Michigan and MUSC to obtain the data used. CT methods were similar to those reported previously[34–37] for 8 participants evaluated at University of Michigan, and the remaining ears (N = 9) at the second institution used alternative CT methods[38–42]. Although these two methods have not been formally compared with one another, they result in similar electrode measures (e.g., medial–lateral distance) used for this study. A previous study examining the relationship between ECAPs and the post-operative CT imaging methods used here found no significant effect of CT analysis type/test location[2]. The post-operative CT metric of interest for the current study was medial–lateral distance, defined as the distance between the electrode and mid-modiolar axis (MMA). We used MMA because our previous study^1^ showed that it more strongly affects ECAP recordings when compared to other CT metrics.

### Speech Recognition in Noise

Speech recognition data were gathered retrospectively from electronic medical records (EMR) of each respective institution. Specifically, we collected all available data for all participants for AzBio sentences [43] presented in a fixed + 10 dBA SNR level (background noise = 20-talker babble), with the target sentences presented at 60 dBA. These data were available for only 22 of the 47 participants in this study.

For speech testing, both the target sentences and noise were presented through a soundfield speaker located at 0 degrees azimuth. All data were collected in a double-walled soundbooth. Because data were collected retrospectively from EMR, the test administrator/scorer varied randomly for each participant. Likewise, the specific list number presented was not always reported for each participant but was chosen at random at the discretion of the clinician. AzBio sentences in noise were chosen to evaluate in this study for the following reasons. First, previous studies have shown that age-related declines in speech understanding unrelated to audibility are most salient in implanted and NH listeners when presented in background noise[7, 28, 44, 45]. Second, ceiling effects are more likely to be present when evaluating speech understanding in quiet; in the present cohort, thirty participants had scores available in the AzBio quiet condition with all participants falling within the range of 59 to 98% (mean = 85.43), thereby potentially constrained by ceiling effects.

### Statistics

Data were analyzed using R Version 4.3.1 [46]. Comparisons between dependent and independent factors were analyzed using linear regression models [lm()] and one-tailed, Pearson product moment correlations [cor.test()]. Multiple regression models were also evaluated using linear regression models [lm()]. Regression model diagnostics were evaluated to assure that assumptions were not violated (‘autoplot’); assumptions are valid for all data presented here. Regression statistics are provided below for each analysis, and corresponding correlational analyses are shown in Table 1. One-way analyses of variance (ANOVA) were used to compare regression models [anova()].

**Table 1:** Correlational matrix showing Pearson correlation coefficients and *p*-values (one-tailed analyses) between all independent and dependent study variables

	**Age (years)**	**AzBio + 10 dB SNR** **(% correct)**	**ECAP N1-P2 latency** **(ms)**	**ECAP IPG effect (µA/nC)**	**Medial–lateral distance (mm)**	**Charge (nC)**
Age (years)	–-	*r* = − 0.582** *p* = *0.002*	*r* = 0.290* *p* = *0.024*	*r* = − 0.335* *p* = *0.011*	*r* = 0.271 *p* = 0.147	*r* = 0.164 *p* = 0.135
AzBio + 10 dB SNR (% correct)		*–-*	*r* = − 0.519** *p* = *0.007*	*r* = 0.464* *p* = *0.015*	*r* = 0.042 *p* = 0.461	*r* = *− *0.124 *p* = 0.292
ECAP N1-P2 latency (ms)			–-	*r* = 0.011 *p* = 0.470	*r* = −0.001 *p* = 0 .498	*r* = *− *0.038 *p* = 0.401
ECAP IPG effect (µA/nC)				–-	*r* = 0.256 *p* = 0.161	*r* = *− *0.221 *p* = 0.071
Medial–lateral distance (mm)					–-	*r* = *− *0.121 *p* = 0.324

## Results

### Effects of Aging on Speech Understanding in Noise

**Fig. 3:**
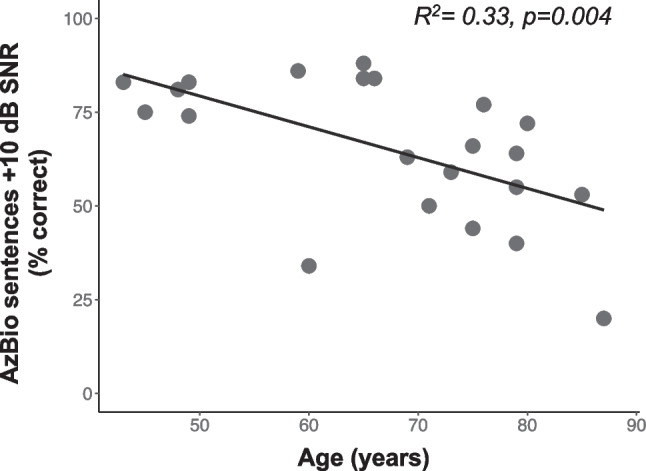
Scatterplot showing the relationship between participant age (x-axis) and speech understanding in noise/AzBio sentences + 10 dB SNR as percent correct (y-axis). Each circle represents a single participant, and the solid black line shows the slope of the regression coefficient

The average score (percent correct) for AzBio sentences in + 10 dB SNR was 65.43% (N = 22, standard deviation = 18.93). The relationship between recipient age and speech understanding in noise is plotted in Fig. 3. Results show that speech understanding in noise, for the 22 subjects assessed in this study, decreased with increasing age (*R*^2^ = 0.33, *p* = 0.004).

### Effects of Aging on the Electrically Evoked Compound Action Potential

**Fig. 4:**
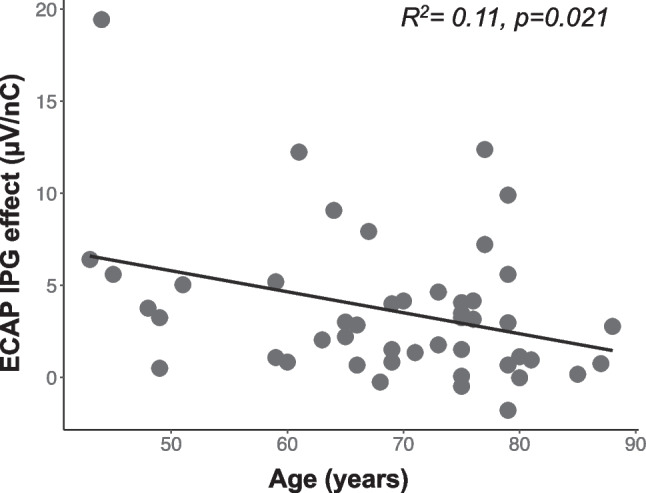
Same as Fig. 3 but showing the relationship between participant age (x-axis) and the ECAP IPG effect for linear slope (µV/nC)

**Fig. 5:**
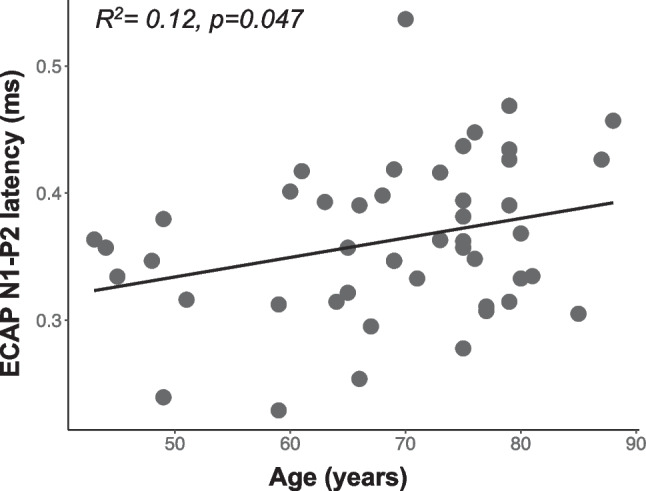
Same as Fig. 3, but showing the relationship between participant age (x-axis) and the ECAP N1-P2 interpeak latency (ms) (y-axis)

The average ECAP-IPG-slope effect value across all participants (N = 47) and electrodes was 3.64 µV/nC (standard deviation = 3.95), and the average ECAP N1-P2 interpeak latency across all participants and electrodes was 0.363 ms (standard deviation = 0.061). The relationships between recipient ages and ECAP measures are shown in Figs. 4 and 5.

**Fig. 6:**
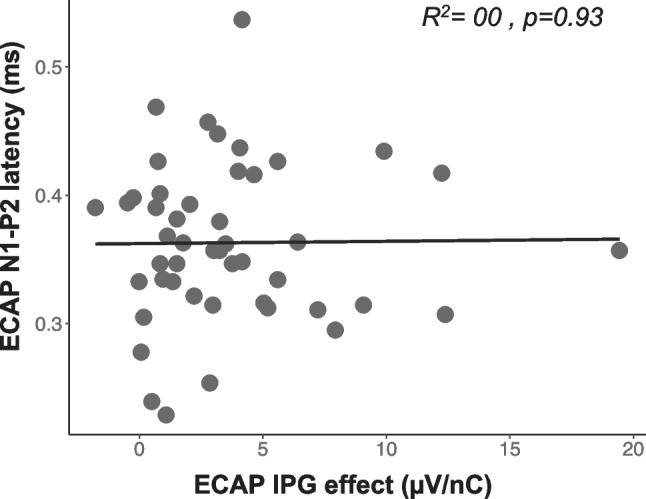
Same as Fig. 3, but showing the lack of relationship between the ECAP IPG effect for linear slope (µV/nC) (x-axis) and ECAP N1-P2 interpeak latency (ms) (y-axis)

These results show that the ECAP IPG effect decreased with increasing age (*R*^2^ = 0.11, *p* = 0.021) (Fig. 4), and the ECAP N1-P2 interpeak latency increased with increasing age (*R*^2^ = 0.12, *p* = 0.047) (Fig. 5). These results suggest that AN density and synchrony decrease with increasing age. However, further analyses showed that these two variables were not significantly correlated with one another (*R*^2^ = 0.00, *p* = 0.93) (Fig. 6).

### Comparison with Medial–Lateral Distance Using Post-operative Computerized Tomography and Stimulation Level (Charge)

The average distance between electrodes and mid-modiolar axis (MMA) across all electrodes and all participants with post-operative CT data (N = 17) was 2.42 mm (standard deviation = 0.375). MMA distance was not significantly correlated with the ECAP IPG effect for slope (*R*^2^ = 0.06, p = 0.321), or the N1-P2 latency (*R*^2^ = 0.00, *p* = 0.99). Similarly, the average MMA distance was not correlated with speech recognition in noise for AzBio sentences (*R*^2^ = 0.00, *p* = 0.92). Lastly, neither AN health measure was significantly correlated with the stimulation level (charge in nC) used to elicit the peak-amplitude response (ECAP IPG effect = *R*^2^ = 0.08, *p* = 0.07; ECAP N1-P2 interpeak latency = *R*^2^ = 0.00, p = 0.801).

### The Relationship Between AN Health Measures, Aging, and Speech Recognition in Noise

**Fig. 7:**
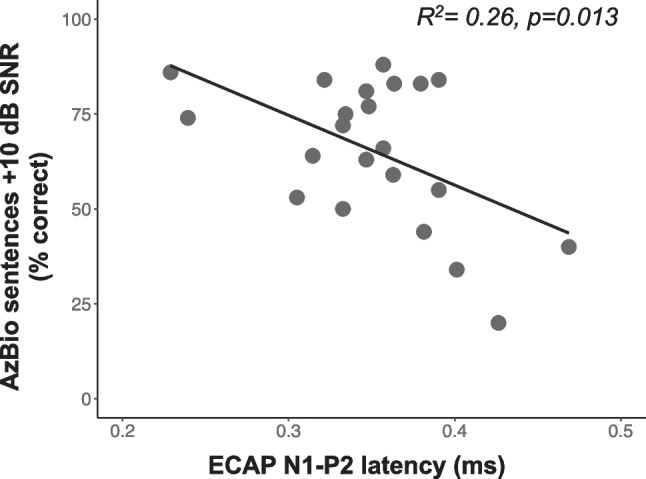
Same as Fig. 3, but showing the relationship between ECAP N1-P2 interpeak latency (ms) and speech understanding in noise/AzBio sentences + 10 dB SNR as percent correct (y-axis)

**Fig. 8:**
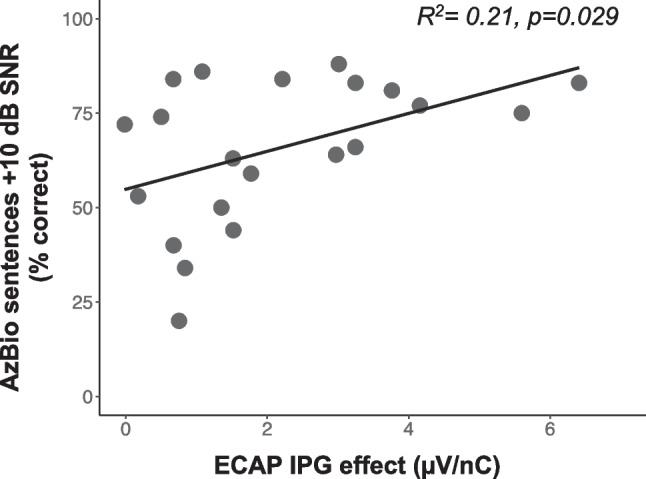
Same as Fig. 3, but showing the relationship between ECAP IPG effect for linear slope (µV/nC) (x-axis) and speech understanding in noise/AzBio sentences + 10 dB SNR as percent correct (y-axis)

Each AN health metric was independently compared to speech recognition in noise understanding. Longer N1-P2 interpeak latencies (greater AN dyssynchrony) were associated with poorer recognition of AzBio sentences in + 10 dB SNR (*R*^2^ = 0.26, *p* = 0.013) (Fig. 7), and higher ECAP IPG effect slope values (greater AN density) were associated with better speech in noise understanding (*R*^2^ = 0.21, *p* = 0.029) (Fig. 8). As noted above these two AN health measures were not significantly correlated with one another (*R*^2^ = 0.00, *p* = 0.930), but both AN measures and speech recognition in noise were associated with aging.

**Fig. 9:**
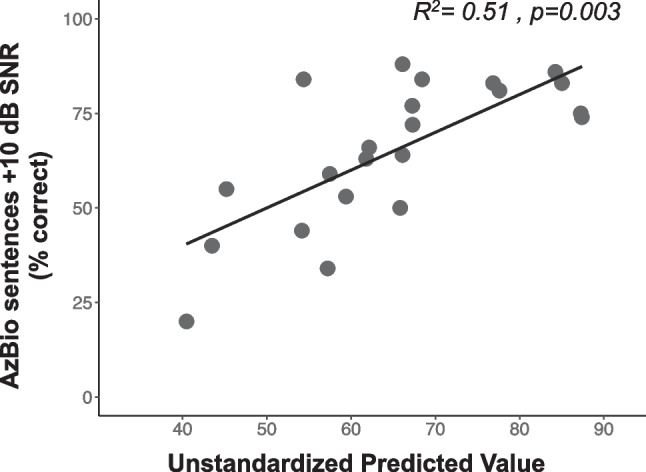
Same as Fig. 3, but displays the results of the multiple regression analysis described as Model 2 in Table 2. This shows the relationship between the unstandardized predicted value obtained from the regression analysis and speech understanding in noise/AzBio sentences + 10 dB SNR as percent correct (y-axis)

In an effort to parse contributing factors to speech recognition, multiple regression models were constructed to determine the extent to which both aging and neural health measures contributed to speech recognition in noise in CI users. Because aging was correlated with speech in noise performance and neural health measures, we sought to determine if neural health measures could help to explain additional variance in speech recognition in noise performance after accounting for listener age. Therefore, the initial model was the same as reported above evaluating the relationship between speech recognition and age (Model 1 = AzBio in noise ~ Age). Then, a second model was constructed by adding N1-P2 interpeak latency (Model 2 = AzBio in noise ~ Age + N1-P2 interpeak latency); this multiple regression model was significant (*R*^2^ = 0.47, *p* = 0.002) and model coefficients are shown in Table 2. An ANOVA was used to compare regression models 1 and 2 to determine if the N1-P2 interpeak latency helped to explain additional variance in speech recognition performance; results showed that adding N1-P2 interpeak latency to the model significantly improved the fit (F = 10.245, *p* = 0.004)). Next, we constructed a third multiple regression model by adding the ECAP IPG effect for slope as a second predictor variable in addition to age (Model 3 = AzBio in noise ~ Age + ECAP IPG Effect for slope). While the overall fit of Model 3 was significant (*R*^2^ = 0.36, *p* = 0.013), the ECAP IPG effect for slope did not contribute significantly to the model and age alone with the only predictive factor (F = 0.775, p = 0.40). These results suggest that poorer AN synchrony, but not AN density, contributed to poorer speech recognition in noise, even after controlling for participant age. Specific results of the regression model are shown in Table 2 and in Fig. 9. Figure 9 plots the unstandardized predicted value derived from Model 2 in Table 2 against speech recognition in noise, showing how both Age and ECAP N1-P2 latency were significantly correlated with speech understanding in noise (*R*^2^ = 0.51, *p* = 0.003). Lastly, it should be noted that additional models using interaction terms were constructed and analyzed, but significant interactions were not present in any model. Therefore, additive models were chosen to best represent the data.

**Table 2:** Multiple linear regression analyses coefficients and model statistics

	**Unstandardized Coefficient**		**Regression statistics**		**Model statistics**		
**Model**		*B*	*Std. Error*	*t value*	*Significance*	*F*	*Significance*
**1**	Age	− 0.412	0.128	− 3.201	0.004	–-	–-
**2**	Age	− 0.664	0.246	− 2.694	0.014	8.460	0.002
	N1-P2 latency	− 134.86	61.86	− 2.180	0.042		
**3**	Age	− 0.66	0.316	− 2.100	0.049	5.422	0.013
	IPG Effect for slope	1.89	2.211	0.858	0.401

## Discussion

The present study examined measures related to neural health that help to explain variance in speech understanding in adult CI recipients. The findings presented here compliment those reported previously in both normal hearing, hearing impaired, and cochlear implanted populations. Specifically, speech recognition performance in adverse listening conditions (e.g., background noise) worsens with increasing age in NH listeners and a similar trend has been shown in individuals who use CIs[7, 23], and as shown in Fig. 3. Age-related declines in speech understanding in NH listeners are thought to be multifactorial[47–51], involving altered peripheral and central neural mechanisms as well as cognitive function. While previous studies show that age-related changes in cognitive function help to account for speech recognition abilities in older CI recipients[52, 53], the findings shown here suggest that age-related declines in AN function also contribute to poorer speech understanding in older CI users. Specifically, a novel but simple measure hypothesized to reflect AN synchrony, *N1-P2 interpeak latency*, correlates with speech recognition performance in noise suggesting that more precise AN synchrony promotes better speech recognition in noise in aging CI listeners.

### Contributions of Aging and other Factors on Auditory Nerve Health

Several studies in humans and animal models show changes to the AN with increasing age. Specifically, the number and/or density of SGN fibers is reduced in older ears and myelin degeneration is present [20, 54–57]; while these changes are thought to contribute to related declines in peripheral hearing sensitivity (e.g., audiometric thresholds), there is variability in AN health of aged cochleae that is somewhat independent of audiometric thresholds. Studies in non-implanted humans show that synchrony of the remaining AN fibers is perhaps more important than the number of remaining fibers[20], which is consistent with the findings we report here in cochlear implanted adults.

Previous studies in CI recipients showed that the density [16, 18, 19, 27] and synchrony [27, 28] of AN fibers helps to predict speech understanding, but aging effects were largely unknown. Shader et al. [58] measured ECAP linear slope, which has been shown to reflect AN density in an animal model[11, 12], and found that ECAP linear slopes were shallower in middle- aged and older CI users compared to younger CI users[58], and this is in agreement with related studies showing a similar trend in older adults[59, 60]. Similarly, we also showed that the ECAP IPG effect for linear slope, also predictive of AN density, decreases with increasing age, suggesting lower SGN density in older CI recipients. While previous studies have explored whether AN temporal resolution decreases with increased age using ECAP recovery functions [61, 62], age-related changes specific to AN dyssynchrony have yet to be defined in CI users. The present study used a novel ECAP measure (N1-P2 interpeak latency) to estimate AN synchrony. Results suggest greater AN dyssynchrony with increasing age. These findings also suggest that age-related changes to AN dyssynchrony observed in non-implanted ears[20] is also present in the electrically stimulated AN. While electrical stimulation of the AN logically results in more synchronous AN activity compared to acoustic stimulation, it seems that this effect is not necessarily sufficient to completely overcome age-related declines in AN synchrony. Importantly, the results of the current study also suggest that the density and synchrony of AN fibers in CI recipients are not completely correlated with one another, suggesting independent underlying age-related mechanisms. While it is true that the ECAP recording does require some degree of synchronicity by definition, results from this study and other recent findings suggest that some dyssynchrony is present, similar to what is observed in non-implanted listeners. Because ECAP peak amplitude is recorded at a high presentation level, it is presumed that ECAP response represents somewhat overlapping neural populations of adjacent electrodes. However, our previous work suggests that even suprathreshold ECAP measures vary across the electrode array within an ear even after controlling for electrode placement thereby suggesting that they largely reflect the response of local AN fibers[1, 2] While both AN density and synchrony were correlated with speech recognition in noise, results presented here suggest that AN synchrony, rather than AN density, is a stronger predictor of speech in noise recognition in older CI users. Lastly, it is uncertain how these two measures of AN health change over a duration of time post implantation. There is evidence from animal models and humans that suprathreshold ECAP peak-amplitude and slope are stable for months or years after cochlear implantation [13, 63], it is not yet known how AN dyssynchrony changes post implantation in CI users, or if it changes as recipients age. Further work is needed to better understand these relationships in a larger cohort of recipients.

In non-implanted ears, several age-related changes to systemic and molecular mechanisms are thought to contribute to age-related declines in AN density [56], while age-related myelin degeneration is thought to contribute to AN dyssynchrony, and can occur before loss of AN fibers [57]. Previous studies have examined whether age-related changes to AN structure and function are solely a natural consequence of aging, or the extent to which other factors such as etiology of hearing loss and noise exposure may play a role. While it can be difficult to completely isolate factors to explore independent effects, it is likely that AN changes observed in older listeners are a consequence of more than one factor[64–67]. For example, noise exposure can alter AN survival and myelination by dysregulation of RNA binding proteins in younger and aged animal models and these studies also suggest that noise exposure can contribute to accelerated loss of AN survival in aged animals [64, 68, 69]. However, it remains difficult to parse out contributing factors to age-related AN structure and function in humans. A recent study by Dias et al. [54] measured the N1 amplitude of the acoustic CAPs in non- implanted humans, which measures AN function akin to ECAP measures in CI users, to assess the independent contributions of noise exposure and aging to AN health in NH listeners. Results showed that age, rather than noise exposure, was the primary contributing factor to age-related changes in CAP amplitudes. An accompanying meta-review of the existing literature in NH humans also shows the same, with age showing a strong association with AN function, and noise exposure played a small or negligible role[54].

Hearing loss etiology is also known to contribute to AN structure and function. Nadol and colleagues measured SGN density in 55 temporal bones with varying etiologies of acquired hearing loss in non-implanted ears and in temporal bones of NH controls[8] and showed that etiology contributes to SGN density, with post-natal viral labyrinthitis showing the lowest density and sudden idiopathic hearing loss showing the highest density. Likewise, common etiologies of congenital hearing loss such as mutations to the GJB2 gene which encode the connexin 26 (Cx 26) protein and enlarged vestibular aqueduct (EVA) result in normal or near normal SGN density [70, 71]. Charcot-Marie-Tooth (CMT) is associated with demyelination in the peripheral nervous system and abnormal AN function [72, 73], and children born with cochlear nerve deficiency (CND) also show abnormal AN function via auditory brainstem response testing[74].

The current study included a cohort of CI recipients with highly varying etiologies and, although not specifically documented or measured, likely varying degrees of noise exposure throughout their lifetime prior to cochlear implantation. Therefore, when examining effects of aging on AN function in CI users it is important to acknowledge that multiple factors could be influencing AN structure and function across the cohort. Likewise, etiology of hearing loss has been shown to contribute to post-operative speech recognition scores in CI users [75, 76], with better outcomes observed for etiologies not directly affecting the condition of the AN. Despite this, the data presented in the current study show that AN structure and function are altered with increased age, and these changes could contribute to poorer speech understanding observed in older CI recipients.

#### The Effects of Electrode Location and Charge on Neural Health Measures

A subset of participants in the present study also underwent advanced post-operative imaging data, which allows for reliable estimates of electrode location measures, including medial–lateral distance and scalar location. While previous studies have shown that scalar location does affect overall speech recognition performance outcomes for CI recipients[42, 77], other studies have shown that scalar location is not significantly associated with ECAP measures that estimate the condition of the AN^9^ and was therefore this factor was not a focus of the present study. Conversely, previous studies showed that several key ECAP measures (e.g., thresholds, peak-amplitude and AGF slope) are affected by the medial–lateral distance defined as the distance in mm between the location of the electrode and the medial wall or mid-modiolus depending on the specific study [1, 2]. These studies also demonstrate that the IPG effect for ECAP slope used in the current study is not significantly affected by medial–lateral distance. Likewise, the ECAP N1-P2 measure of AN synchrony (N1-P2 interpeak latency) explored here also seems to be independent of medial–lateral distance (Table 1). Furthermore, Table 1 shows that the N1-P2 interpeak latency is not significantly correlated with average charge (nc) values within an ear. This is also important to consider given that N1-P2 values were taken at the peak amplitude of responses across the array, and one might expect that higher peak amplitude charge might influence N1-P2 latency values (e.g., higher charge associated with shorter latencies).

#### Comparison of N1-P2 Interpeak Latency with other AN Synchrony Measures

The current study uses N1-P2 interpeak latency to estimate AN synchrony in CI users. This method is advantageous because it uses routine clinical methods and commercially available hardware and software without significant post-processing of data but is seemingly able to capture the trial-by-trial fluctuations in the N1 and P2 components. In non-implanted listeners, AN synchrony can be estimated with either the PLV (also known as inter-trial coherence) of the N1 of the CAP, or with half-width[20, 22] latency of the N1 component, a measure analogous to interpeak latency used here. PLV in NH listeners is a valid method for assessing synchrony, which is associated with myelin in animal models[29].

PLV is calculated in the frequency domain; the acoustically evoked AN response occurs within 1–2 ms following sound onset, resulting in phase-locking that peaks around 1500 Hz. PLV has been used to estimate AN synchrony in CI users as well[28], but there are significant challenges in its calculation due to the timing of the ECAP, the maximum sampling rate of CIs, and the number of samples recorded in an ECAP. In an electrically evoked AN response, the N1 peak occurs much earlier (0.2–0.4 ms) compared to acoustic stimulation of non-implanted humans. There is a direct relationship between the lowest frequency that can be resolved and the duration of the recording window, such that the lower the frequency the longer the time window that is needed. For most commercially available software/hardware, the recording window is insufficient in duration to allow for recording of PLV in CI users, and adequate frequency resolution for the PLV in CI users would require ECAP recordings over a longer time period than is routinely clinically performed, or furthermore, are likely not possible given current limitations of clinical hardware and software. Moreover, PLV is typically assessed relative to a baseline period, which is also not present in standard clinical recordings. While other studies in NH listeners have also used ECAP N1 half-width instead of PLV to estimate AN synchrony [22] this is also challenging in CI ECAP recordings. Half-width latency calculation traditionally relies on adequate resolution and identification of the P1 peak just prior to the N1, which is possible in acoustic CAPs. However, in ECAP recordings the P1-N1 complex is not always clearly visible making it difficult to accurately calculate the half-width latency as a proxy for AN synchrony in CI users. The method presented here is simple, and easily measured using standard recording parameters available in commercially available materials.

#### Implications for CI Outcomes in Older Listeners

While previous studies have shown that AN density can be predictive of speech understanding in CI users, the results of the present study suggest that AN synchrony rather than AN density is more important for speech understanding in noise. Results also suggest that poorer AN synchrony in older adults may contribute to declining speech understanding in older CI recipients. These data are in keeping with studies in non-implanted listeners that show that poorer AN synchrony leads to poorer speech understanding in noise [20]. While speech understanding in noise generally improves at least slightly in older CI users when comparing pre-to-post op performance measures, the results of the current study have important implications for expectations following cochlear implantation in older listeners. Specifically, if electrical stimulation of the AN is not necessarily sufficient to overcome dyssynchrony as suggested by our results, and if AN dyssynchrony is a contributing factor to poorer speech in noise understanding in older CI users, then AN dyssynchrony would continue to contribute to poorer speech understanding once patients are implanted. As CI candidacy continues to expand and insurance criteria for CIs are largely based on perception of sentences in noise, it is important to understand and consider the limitations of cochlear implantation in an aging ear. The condition of the AN could be a limiting factor in the success of CI outcomes in older listeners, and further work is needed to better understand these limitations and maximize outcomes in older CI recipients.

## Data Availability

The data that support the findings of this study are not openly available but are available from the corresponding author upon reasonable request. Data are located in controlled access data storage at the Medical University of South Carolina.

## Code Availability

MATLAB coding used in the current study to calculate ECAP metrics are not openly available, but are available from the corresponding author upon reasonable request.
